# AAV-Delivered Antibody Mediates Significant Protective Effects against SIVmac239 Challenge in the Absence of Neutralizing Activity

**DOI:** 10.1371/journal.ppat.1005090

**Published:** 2015-08-06

**Authors:** Sebastian P. Fuchs, José M. Martinez-Navio, Michael Piatak, Jeffrey D. Lifson, Guangping Gao, Ronald C. Desrosiers

**Affiliations:** 1 Department of Pathology, Miller School of Medicine, University of Miami, Miami, Florida, United States of America; 2 Institut für Klinische und Molekulare Virologie, Friedrich-Alexander-Universität Erlangen-Nürnberg, Erlangen, Germany; 3 AIDS and Cancer Virus Program, Leidos Biomedical Research, Inc., Frederick National Laboratory, Frederick, Maryland, United States of America; 4 Gene Therapy Center, University of Massachusetts Medical School, Worcester, Massachusetts, United States of America; University of Zurich, SWITZERLAND

## Abstract

Long-term delivery of potent broadly-neutralizing antibodies is a promising approach for the prevention of HIV-1 infection. We used AAV vector intramuscularly to deliver anti-SIV monoclonal antibodies (mAbs) in IgG1 form to rhesus monkeys. Persisting levels of delivered mAb as high as 270 μg/ml were achieved. However, host antibody responses to the delivered antibody were observed in 9 of the 12 monkeys and these appeared to limit the concentration of delivered antibody that could be achieved. This is reflected in the wide range of delivered mAb concentrations that were achieved: 1–270 μg/ml. Following repeated, marginal dose, intravenous challenge with the difficult-to-neutralize SIVmac239, the six monkeys in the AAV-5L7 IgG1 mAb group showed clear protective effects despite the absence of detectable neutralizing activity against the challenge virus. The protective effects included: lowering of viral load at peak height; lowering of viral load at set point; delay in the time to peak viral load from the time of the infectious virus exposure. All of these effects were statistically significant. In addition, the monkey with the highest level of delivered 5L7 mAb completely resisted six successive SIVmac239 i.v. challenges, including a final challenge with a dose of 10 i.v. infectious units. Our results demonstrate the continued promise of this approach for the prevention of HIV-1 infection in people. However, the problem of anti-antibody responses will need to be understood and overcome for the promise of this approach to be effectively realized.

## Author Summary

AAV-mediated antibody delivery represents a promising alternative to classical vaccine approaches for the prevention of HIV/AIDS in humans. We used recombinant adeno-associated viral vectors (rAAV) as gene carrier to deliver anti-SIV (simian immunodeficiency virus) antibodies to monkeys. This non-classical immunization approach is independent of the host immune system and the AAV-transferred gene into muscle cells produces antibodies with known specificities. Our goal was to determine what serum levels of delivered antibodies can be achieved by using two different vector strategies, whether the delivered antibodies provoke an immune response, and to what degree the delivered non-neutralizing antibodies have the ability to protect against SIV virus challenge. This approach allowed for sustained levels of delivered antibodies in serum and exhibited significant protective effects against highly pathogenic SIVmac239 challenge. However, immune responses limited the concentration of circulating antibodies that could be achieved. This problem will need to be understood and overcome for the promise of this approach to be effectively realized.

## Introduction

There are good reasons for believing that development of an effective preventive vaccine against HIV-1 is going to be a very difficult task. HIV-1 has evolved a variety of immune evasion strategies that allow continuous virus replication in the face of apparently strong host immune responses, both cellular and humoral [1,2]. For a vaccine to be effective it will likely need to either completely block the initial infection or to provide immune responses that are more effective than those resulting from infection.

One promising, creative approach is to use adeno-associated virus (AAV) vector to deliver antibodies with potent and broadly neutralizing activity [3–7]. This method is an alternative to classical immunization and independent of the host immune system. Proteins delivered by AAV vector can persist at stable levels for years [8,9], and AAV vectors have proven to be safe and effective in human gene therapy trials against a number of diseases [10–13]. More than a dozen potent broadly neutralizing anti-HIV-1 monoclonal antibodies have already been characterized; they are human-derived, they can effectively neutralize more than 90% of circulating HIV-1 strains, and they represent a range of different specificities [14–21].

In a previous pioneering study performed in monkeys, anti-SIV immunoadhesins (antibody-like molecules) were delivered by AAV vector and a reasonable percentage of the monkeys exhibited apparent sterilizing immunity against intravenous (i.v.) challenge by SIVmac316 [4]. However, SIVmac316 is an easy-to-neutralize SIV strain, one third of the immunized monkeys developed antibody responses (anti-anti) to the delivered immunoadhesin, and monkeys with anti-anti responses were not protected against the challenge. It was not clear from that initial study to what extent anti-anti responses may have resulted from delivery of immunoadhesins, which do not represent an authentic IgG structure. We decided to build upon these studies by using AAV to deliver authentic IgG and to examine protection against the difficult-to-neutralize strain SIVmac239. At the time these studies were initiated, there were no monoclonal antibodies available that could neutralize SIVmac239. We thus converted the previously reported immunoadhesins 4L6 and 5L7 [4] into authentic IgG versions. These anti-SIV mAbs in IgG1 form contain only authentic rhesus IgG sequences. In this study we describe the AAV-mediated delivery of these full-length immunoglobulins to rhesus macaques and the protective effects of the non-neutralizing antibody 5L7 IgG1 against SIVmac239 challenge.

## Results

### AAV-mediated delivery of mAbs

Two fundamentally different types of AAV systems are available for vector-mediated delivery: single-stranded (ssAAV) *vs*. self-complementary (scAAV) [22]. scAAV has been reported to achieve considerably higher levels of transgene expression [23,24] but suffers from the drawback of not being able to accommodate sufficient genetic information for the expression of both heavy (H) and light (L) chains needed for authentic IgG [25]. We thus developed two strategies for AAV-mediated synthesis of authentic IgG for comparison purposes: ssAAV for the synthesis of matched H and L chains from a single vector *vs*. scAAV for the synthesis of H and L chains from separate vectors, an approach that we will call the two vector approach. The logic behind the two vector approach is that such a high quantity of AAV vector particles is inoculated into a highly localized area of muscle that it seems likely that many, perhaps even a majority, of cells will take up multiple AAV particles. We sought to deliver authentic IgG versions of the previously described monoclonal Fabs 346-16h and 347-23h [26] in order to allow comparison to the results of Johnson *et al*. that utilized immunoadhesins with these binding specificities (called 4L6 and 5L7, respectively) [4]. Since SIVmac239 was planned for challenge, this also allowed investigation of protective effects in the absence of virus-neutralizing activity. We employed IgG1 sequences in order to maximize antibody-dependent cellular cytotoxicity (ADCC) activity and other effector functions. We used AAV serotype 1 for intramuscular expression. The constructs shown in (S1 Fig) were demonstrated to express authentic rhesus IgG with expected properties prior to the initiation of monkey experiments.

**Fig 1:**
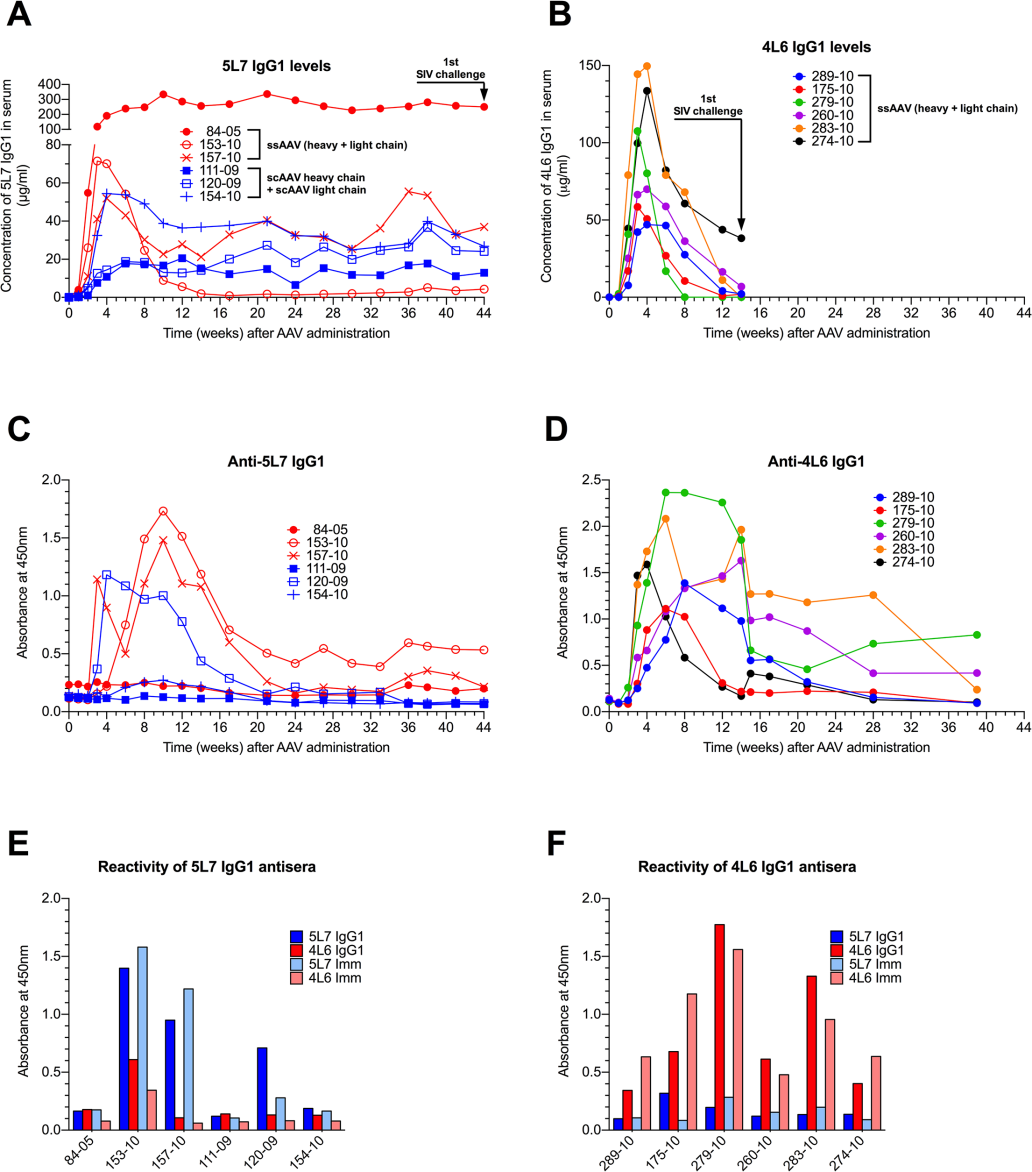
Serum concentration of the IgG1 mAbs 4L6 and 5L7, and emerging anti-mAb responses following recombinant AAV administration. Levels of produced antibodies were measured in sera from immunized animals over time by a gp140 capture ELISA. SIV challenge is indicated by arrows. (**A**) Animals that received rAAV-5L7 IgG1. 84–05, 153–10 and 157–10 were given one ssAAV vector expressing both heavy and light chain of the antibody 5L7 IgG1. 111–09, 120–09 and 154–10 were given an equal mixture of two scAAV vectors expressing either heavy chain or light chain of the antibody 5L7 IgG1. (**B**) Animals that received rAAV-4L6 IgG1. All six animals were given one ssAAV vector expressing both heavy and light chain of the antibody 4L6 IgG1. (**C** and **D**) Antibody responses to 5L7 IgG1 and 4L6 IgG1, respectively, following recombinant AAV administration. Humoral immune responses were measured in sera from immunized animals over time by ELISA. The reactivity of serum was tested against homologous purified protein using a conjugated anti-lambda secondary antibody for detection since both 5L7 IgG1 and 4L6 IgG1 bear a kappa light chain. (**E**) Reactivity of sera from 5L7 IgG1 recipients to purified mAbs and immunoadhesins at week 10 after AAV administration. (**F**) Reactivity of sera from 4L6 IgG1 animals to purified mAbs and immunoadhesins at week 6 after AAV administration.

In our first monkey study, three AAV1-negative monkeys were inoculated intramuscularly with 1.6 x 10^13^ ssAAV vector particles expressing both heavy and light chains of the authentic rhesus monkey IgG1 monoclonal antibody 5L7 (ssAAV-5L7 IgG1). On the same date, three AAV1-negative monkeys were inoculated with 0.8 x 10^13^ scAAV vector particles expressing 5L7 IgG1 heavy chain and 0.8 x 10^13^ scAAV vector particles expressing 5L7 kappa light chain (scAAV-5L7 IgG1 H + scAAV-5L7 kappa L). The levels of delivered 5L7 mAb in plasma were measured by a gp140 ELISA and purified mAb as standard. Considerable monkey-to-monkey variation was observed in the levels of 5L7 IgG1 that were achieved (Fig 1A). However, the levels in each individual monkey remained stable over the course of months leading up to the time of challenge. There were no clear differences in the persisting levels of 5L7 IgG1 mAb associated with the one vector *vs*. two vector delivery systems. While monkey 153–10 in the one vector group achieved persisting levels of 5L7 IgG1 mAb of only 1–5 μg/ml, monkey 84–05, also in the one vector group, achieved remarkable levels of approximately 270 μg/ml that have persisted now for more than two years. Three of the six monkeys in this first study had clear antibody responses to the delivered 5L7 IgG1 mAb (Fig 1C and S2 Fig). Monkey 153–10 with the lowest level of persisting 5L7 mAb had the strongest anti-anti response, while monkey 84–05 with the highest level of persisting 5L7 mAb had no detectable anti-anti response. The other two monkeys without detectable anti-anti responses (111–09 and 154–10) were in the two vector scAAV group.

In our second monkey study, we used the one vector ssAAV approach to deliver the 4L6 mAb in IgG1 form to six AAV1-negative monkeys. In this experiment, the levels of delivered 4L6 mAb peaked 3–4 weeks after administration and then fell precipitously (Fig 1B). All six of the recombinant AAV-4L6 recipients had antibody responses to the delivered 4L6 mAb whose appearance coincided with the precipitous decline in 4L6 mAb levels (Fig 1D and S3 Fig). The anti-anti responses in the 5L7 IgG1 recipients (Fig 1C) were largely specific for 5L7 while the anti-anti responses in the 4L6 IgG1 recipients (Fig 1D) were largely specific for 4L6; since the 5L7 and 4L6 IgG1s have the exact same constant regions, these results indicate that the responses were mostly directed to the variable domains (Fig 1E and 1F). By week 14, the time of SIV challenge, the levels of 4L6 mAb in two of the recipients (279–10 and 283–10) had dropped to undetectable levels, while the levels in the other four monkeys had dropped to 1–38 μg/ml (Fig 1B).

### SIV challenge of monkeys

Six appropriately-matched control rhesus monkeys were enrolled in the challenge phase (S1 Table). Repeated, marginal dose, intravenous challenges were simultaneously initiated to control monkeys, to the 5L7 mAb group 44 weeks after their administration of recombinant AAV-5L7 IgG1, and to the 4L6 mAb group 14 weeks after their administration of recombinant AAV-4L6 IgG1. Administrations were performed every three weeks such that administrations were stopped when a monkey became infected from the previous exposure. A PBMC-grown stock of cloned SIVmac239 was used, one that has been carefully titered previously by the i.v. route in monkeys and has been used extensively by numerous investigators for controlled dose challenges [27–35]. One i.v. animal infectious dose was used through the first four administrations, 2 i.v. infectious doses for the fifth administration, and 10 i.v. infectious doses for the sixth administration.

**Fig 2:**
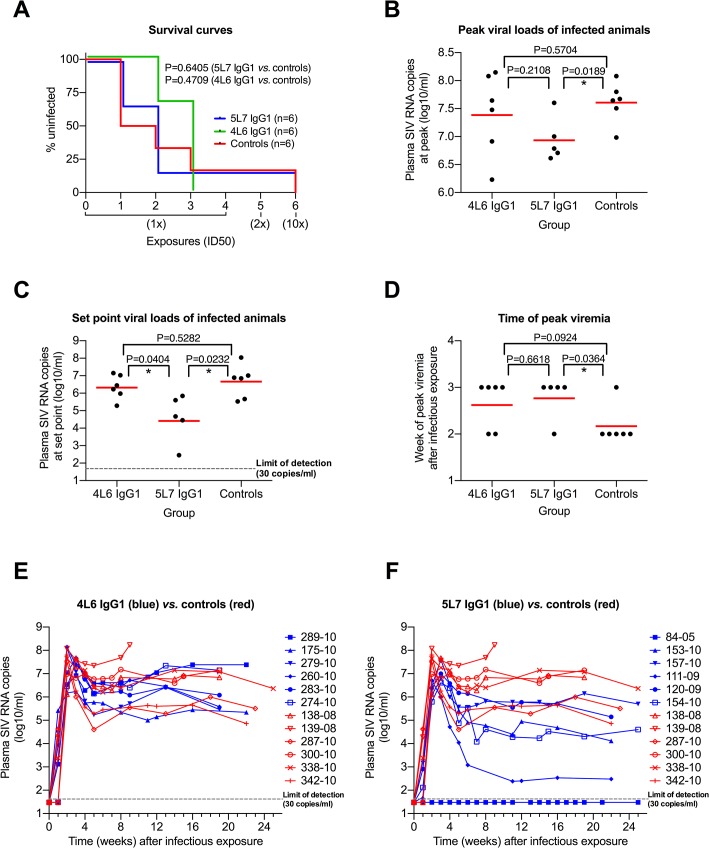
Repeated low-dose SIVmac239 challenge of AAV-immunized and control animals. (**A**) Kaplan-Meier analysis of the three test groups. The percentage of animals remaining uninfected is plotted against the number of SIV exposures. SIV virus challenges were conducted in 3-week intervals. Four low-dose challenges with one half-maximal infectious dose (1x ID50) were followed by a two ID50 (2x) and a subsequent ten ID50 (10x) challenge. The survival curves are not significantly different (Mantel-Cox test). (**B**) Peak viral load comparison. The geometric mean (red line) is significantly different in the 5L7 IgG1 group compared to the control group (two-tailed, unpaired *t* test). (**C**) Set-point viral load analysis. All values within one group between weeks 8 and 25 were averaged, the resulting values were logarithmized (log10) and compared to each other. The geometric mean of the 5L7 IgG1 group differs significantly from the other two groups (two-tailed, unpaired *t* test). (**D**) Time of peak viremia. Most of the immunized animals show peak viremia at week 3, while 5 of 6 controls peak at week 2. (**E** and **F**) Viral loads *in vivo* after infectious exposure with SIVmac239. Viral loads in plasma of AAV animals (blue) and controls (red) measured as SIV RNA genome equivalent per ml are shown as a function of time since the infectious exposure. Control animal 139–08 was a rapid progressor and had to be sacrificed after 9 weeks due to AIDS-related symptoms. Test animal 84–05 remained uninfected after 6 SIV challenges.

Only one monkey of the 18 resisted all six challenges: monkey 84–05 with the highest levels of delivered mAb (averaging approximately 270 μg/ml of the 5L7 mAb through all of the challenges) (Fig 2A). Dozens of control monkeys have been challenged with the 10x dose of this exact same stock in numerous previous studies and not a single monkey has been refractory to infection [27–35]. The absence of anti-gp41 antibody responses in 84–05 even following the 10x challenge is consistent with the apparent sterilizing immunity in this animal (S4 Fig). Otherwise, no significant difference in the acquisition of SIV infection was observed among the test *vs*. control monkeys (Fig 2A).

Given that levels of delivered 4L6 IgG1 mAb were driven to low or undetectable levels by the time of first challenge in most monkeys in this group, it is not surprising that plasma virion SIV RNA levels in the 4L6 mAb group as a function of weeks following the infectious exposure were not significantly different from control monkeys, neither at peak viremia nor during chronic phase infection (Fig 2B and 2C; Fig 2E). However, SIV plasma RNA loads in the 5L7 mAb group were significantly lower than those in the control group both at peak viremia (Δ of 0.67 logs) and during chronic phase infection (Δ of 2.06 logs) (Fig 2B and 2C; Fig 2F). The differences were significant whether or not the undetectable viral loads of monkey 84–05 were included in the analyses. The viral loads in the 5L7 mAb group at set point were also significantly lower than those in the 4L6 mAb group (Fig 2C). The duration in weeks from the time of the infectious exposure to peak plasma viral loads was also significantly different between the 5L7 mAb group and the control group (Fig 2D). While there is a suggestion that viral loads at peak height were inversely correlated with the levels of delivered 5L7 mAb, this correlation did not reach statistical significance (S5 Fig). There was no obvious association of measured 4L6 IgG1 levels in serum at the time of infectious exposure with the time to peak viremia (S6 Fig).

### Effector functions

**Fig 3:**
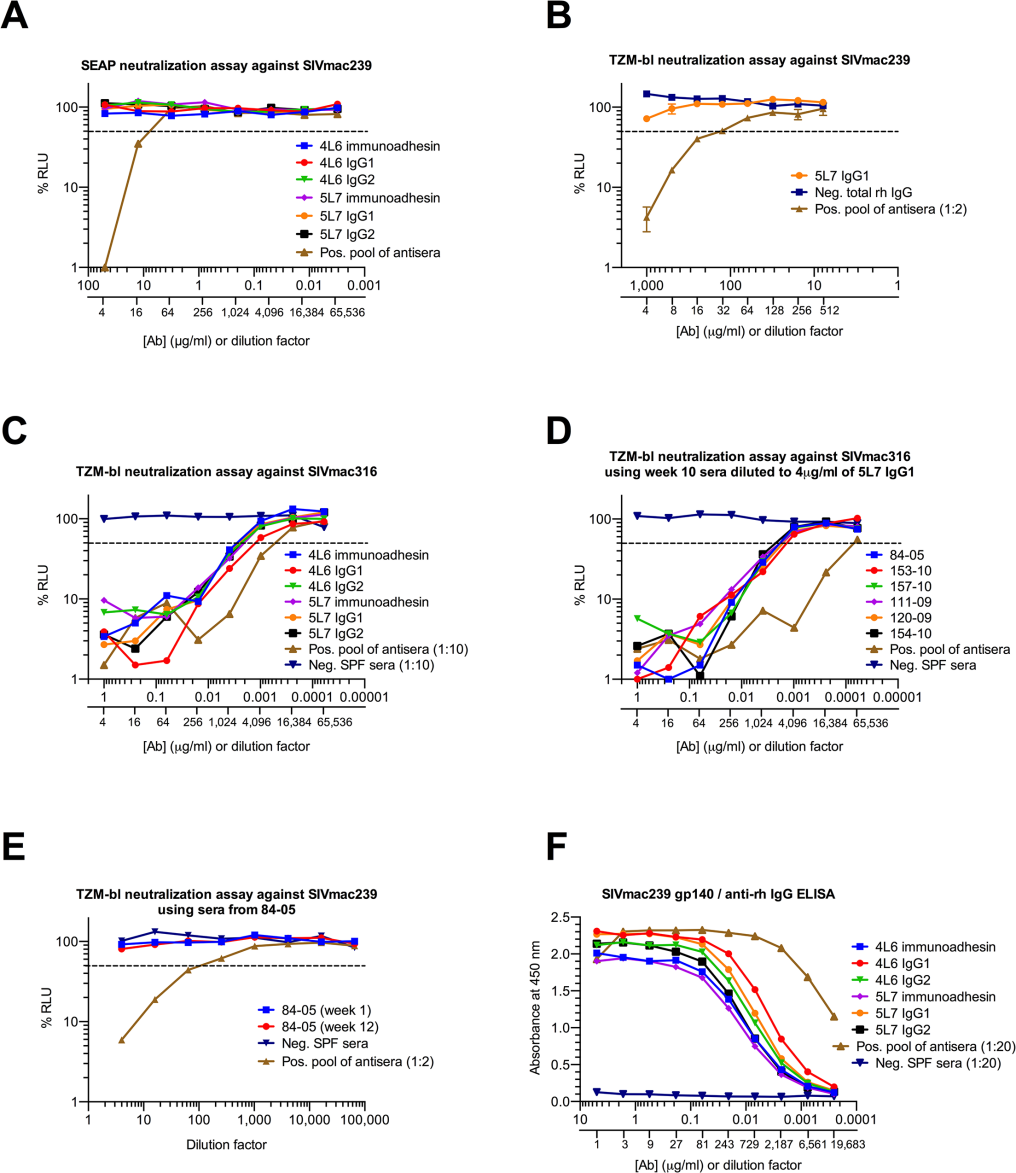
Neutralization of SIV and binding of purified proteins *in vitro*. The dashed line indicates 50% RLU (relative light units) representing 50% neutralization activity against the tested SIV strain. Lowest RLU indicates highest neutralization. Binding of purified proteins was tested by a SIVmac239 gp140 ELISA, high absorbance indicates high binding. SPF serum (specific pathogen free) from naïve animals was used as negative control, a pool of antisera from SIV-infected animals served as positive control. (**A**) None of the antibodies or immunoadhesins showed neutralization against the resistant virus strain SIVmac239 tested at 50 μg/ml. Similar results were obtained in both the SEAP and TZMbl assays. (**B**) Neutralization curve of SIVmac239 with 5L7 IgG1 starting at 1mg/ml. (**C**) All of the tested antibodies and immunoadhesins showed equivalent neutralization activity against SIVmac316 with an average IC50 (half-maximal inhibitory concentration) of 0.002 μg/ml. (**D**) Animal sera from week 10 after rAAV administration (see Fig 1A) were diluted to 4 μg/ml based on previous ELISA quantitations, and tested for neutralizing activity against SIVmac316. The average IC50 measured corresponds to the IC50 of the purified proteins. (**E**) Animal sera of 84–05 from weeks 1 and 12 after AAV administration lacked detectable neutralizing activity against SIVmac239. (**F**) Antibodies and immunoadhesins were tested for their ability to bind SIVmac239 gp140. All of the antibody constructs showed equivalent binding activity as determined by absorbance at 450 nm.

Both the 5L7 and the 4L6 mAb IgG1s lack detectable neutralizing activity against the challenge strain SIVmac239 (Fig 3A). In fact, even at 1 mg/ml of the 5L7 mAb IgG1, 50% neutralization of SIVmac239 was not achieved (Fig 3B). However, both the 5L7 and 4L6 mAbs had neutralizing activity against the neutralization-sensitive derivative of SIVmac239 called SIVmac316 (Fig 3C). The half-maximal inhibitory concentrations (IC50s) against SIVmac316 were 0.0015 μg/ml (4L6 IgG1) and 0.003 μg/ml (5L7 IgG1), which are consistent with previously described IC50s for Fab molecule and immunoadhesin versions of these mAbs [4,26]. The concentrations of mAb in sera shown in (Fig 1A) were measured biochemically with a gp140 ELISA using varying amounts of purified mAb produced by transfection of 293T cells in culture as standards. In order to validate the biochemically measured mAb concentrations and to confirm the expected neutralizing activity in serum against SIVmac316, serum samples were diluted to contain a starting concentration of 4 μg/ml 5L7 mAb based on the biochemically-measured concentration and neutralizing activity against SIVmac316 was measured (Fig 3D). The neutralizing activity in serum agreed spot-on with the biochemically-measured concentration of the 5L7 mAb for all six samples. As expected, sera from 84–05 lacked detectable neutralizing activity against SIVmac239 (Fig 3E). HEK 293T-expressed purified immunoadhesins and mAbs showed equivalent binding to SIVmac239 gp140 as determined by ELISA (Fig 3F).

**Fig 4:**
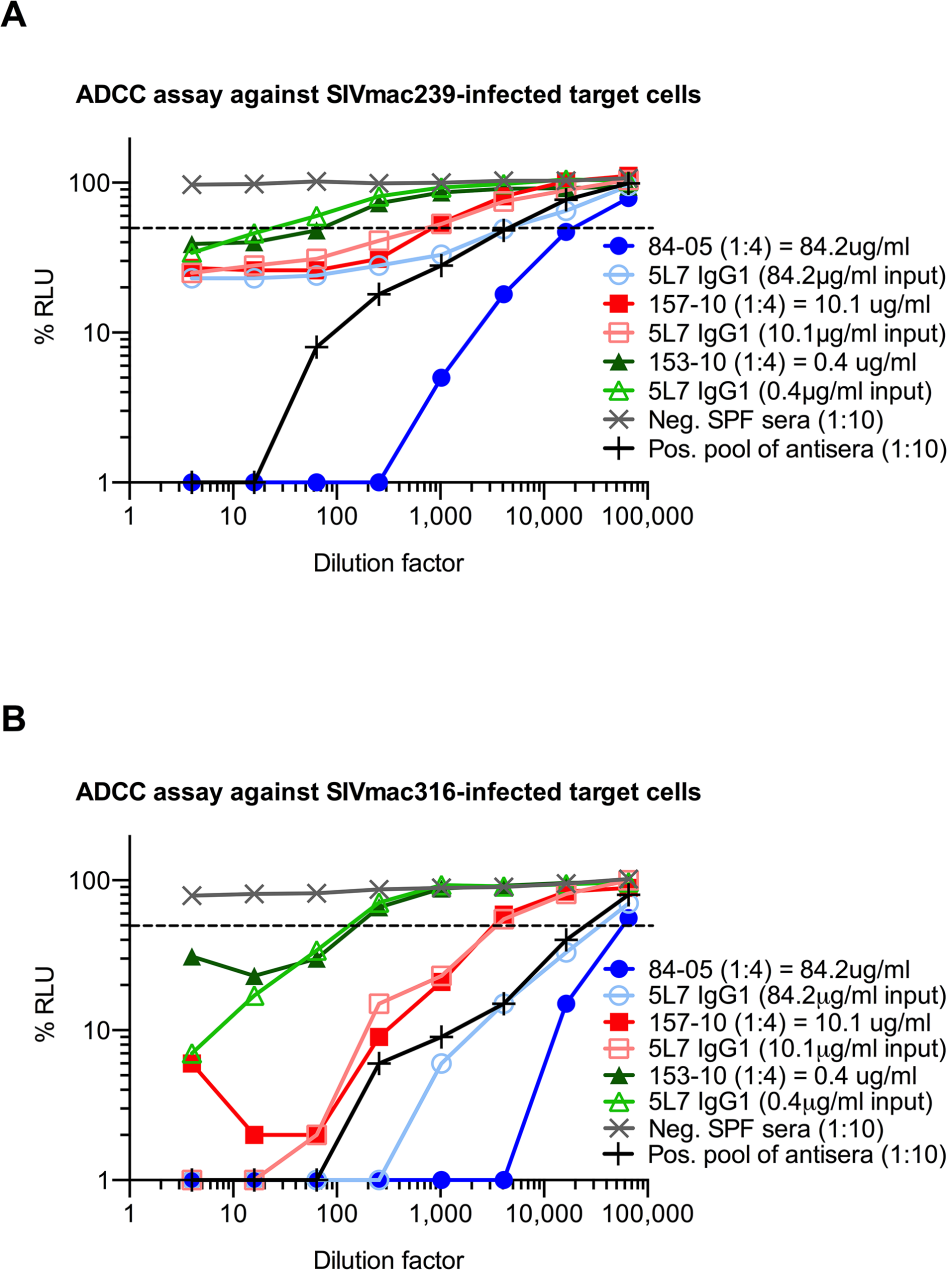
ADCC activity of purified proteins and sera against SIV-infected target cells *in vitro*. The dashed line indicates 50% RLU (relative light units) or 50% ADCC activity against SIV-infected target cells. ADCC was measured by the luciferase activity in SIV-infected cells after an 8 to 10 h incubation in the presence of a macaque CD16^+^ NK cell line and a serial dilution of antibodies or animal sera. The loss of RLU indicates the loss of virus-infected cells during the 8 to 10 h incubation period and represents a high ADCC activity. Purified 5L7 IgG1 was diluted to match the equivalent 5L7 IgG1 concentration of 5L7 IgG1-containing animal sera from week 21 post AAV administration, and compared for mediating ADCC towards (**A**) SIVmac239-infected cells and (**B**) SIVmac316-infected cells. The ADCC activities of sera from 153–10 and 157–10 overlapped with the purified protein at their corresponding concentration. 5L7 IgG1-containing animal serum from 84–05 mediated superior ADCC activity compared to purified 5L7 IgG1 at equivalent concentration of this antibody.

With this information in hand, we proceeded to measure ADCC activity in pre-challenge serum samples relative to the biochemically measured 5L7 mAb concentrations in them. We used an assay that measures natural killer (NK) cell-mediated ADCC activity of mAb or serum independent of neutralization or complement activity [36]. The assay uses a NK cell line that expresses macaque CD16 as effector cell and a CD4^+^ T cell line that expresses luciferase upon SIV infection as target. 5L7 IgG1 purified from 293T transfection was diluted to match the starting concentration of 5L7 IgG1 in the serum sample to which it was being compared. Serum from 84–05 was unusual in that it had markedly higher ADCC activity than the equivalent concentration of purified 5L7 IgG1 (Fig 4A and 4B). Pre-AAV serum from 84–05 contained no measurable activity capable of enhancing the ADCC activity of purified 5L7 IgG1 (S7 Fig). Four of the five remaining animals in the group had ADCC activity in their serum samples comparable to that of purified 5L7 IgG1 at equivalent concentrations (Fig 4A and 4B; S8 Fig). Serum from monkey 111–09, the monkey with the lowest post-challenge viremia, also had somewhat higher ADCC activity compared to purified 5L7 IgG1 at equivalent concentrations (S8 Fig).

## Discussion

Given the current availability of a spectacular array of human mAbs with potent broadly-neutralizing activity against HIV-1, it has become possible to envision prevention scenarios in which cocktails of such antibodies could be used to provide a sterilizing barrier to infection against the vast majority of HIV-1 strains circulating in the population. However, it is not practically feasible to consider passive delivery of purified mAbs over a period of years for prevention purposes. AAV vectors are ideally suited for this purpose. The only protein expressed from AAV vectors is derived from the inserted transgene put into it. As long as the protein is viewed as self, protein expression can continue for prolonged periods. In fact, there are already examples where proteins delivered by AAV vector have persisted at stable levels for years [4,8,9,37], including animal 84–05 in our study. Since muscle cells exhibit little or no turnover, they are a preferred source for long-term delivery purposes. Little or no integration of AAV vector DNA into host genome sequences has been observed [38–40] and AAV vectors have proven safe when used in clinical settings to reverse hereditary absence of gene function [10–13].

The molecularly cloned SIVmac239 is a difficult-to-neutralize strain with many of the characteristics of a primary isolate. It has been extensively used as a challenge strain to gauge efficacy of vaccine approaches in monkeys, and it has generally proven quite difficult to achieve protection against [29,30,41–43]. Being a molecular clone, it avoids complications of interpretation that may arise when a heterogeneous mixture of sequences in an uncloned virus stock are used and when differences in neutralization sensitivity/resistance may exist within the mixture [44,45]. The presence of AAV-delivered 5L7 mAb in our experiments significantly delayed the time to peak viral loads and significantly lowered SIVmac239 viral loads both at peak viremia and at viral load set point. The lowering of viral load set points in the 5L7 mAb group was also significantly different from the 4L6 mAb group, lending additional support to the significance of the protective effects. It is also likely that the lack of SIVmac239 acquisition in monkey 84–05 is also a real effect of the high concentration of 5L7 mAb in this animal. The 10x (6^th^) challenge of this monkey utilized the same dose of the same stock that has been used previously in dozens of control monkeys without a failure of infection acquisition [27–35]. The protective effects in the 5L7 mAb group occurred in the absence of any detectable neutralizing activity of the 5L7 mAb against the SIVmac239 molecular clone even out to 1 mg/ml. To our knowledge, this is the first demonstration of significant protective effects of a mAb in the absence of an ability to neutralize the challenge virus [46,47].

Recent studies have emphasized the importance of Fc-mediated effector functions in contributing to the protection afforded by mAbs capable of neutralizing the SHIV used for challenge [48–52]. These studies modulated the Fc-mediated effector functions of a neutralizing mAb up or down in order to examine the effects on protective capacity. Some vaccine studies have also found associations of ADCC activity with protective efficacy [53–57]. Effector functions potentially contributing to the protective effects of the 5L7 IgG1 observed in our current study include but are not limited to ADCC activity and complement-mediated virolysis. Along these lines, it is fascinating that the 5L7 mAb circulating in monkey 84–05 was not only present at extraordinarily high levels, it had extraordinarily high ADCC activity on a per μg basis. Because of the nature of the ADCC assay that was used, it seems likely that the extraordinarily high ADCC activity in the serum of this one animal results from differences in post-translational modifications of the 5L7 mAb. Decreased core fucosylation or terminal sialylation have been shown to dramatically increase Fc-mediated ADCC activity [58–62].

One question addressed in our current studies was whether the anti-anti responses to the immunoadhesin constructs observed in Johnson *et al*. [4] could be avoided by delivery of authentic IgG. The answer is a clear no. Immune responses to the delivered IgG1 antibody described here were readily detected in 9 of the 12 test monkeys and these appeared to limit the levels of delivered antibody that could be achieved. This is especially true of the 4L6 group in which the levels of delivered 4L6 IgG1 mAb dropped precipitously coincident with the appearance of the anti-anti responses. In separate studies, we have observed strong antibody responses in rhesus monkeys to AAV-delivered, rhesusized anti-HIV-1 mAbs in 24 of 24 opportunities using a ssAAV1 vector design identical to the one used for the studies described here. This contrasts strikingly with studies in humans in which 13 sequential administrations of 1g of anti-HIV mAb failed to elicit anti-anti responses to the three different antibodies that were used in each of the 12 test subjects [63,64]. With our 5L7 and 4L6 experiments, it is possible that a lack of natural pairing of IgG heavy and light chains contributed to their immunogenicity. With our anti-HIV experiments referred to above, it is possible that the failure to rhesusize the variable regions contributed to their immunogenicity. Nonetheless, our results serve as a warning signal for the potential of immunogenicity problems when AAV is used to deliver anti-HIV mAbs in human trials.

## Materials and Methods

### Recombinant AAV vector constructs

Coding sequences (heavy chain, light chain or bicistronic) were designed *in silico*, codon-optimized and gene-synthesized (Genscript). 4L6 and 5L7 immunoadhesin sequences[4] served as a template and full-length antibodies were constructed by adding CH1 domain and CL domain of rhesus IgG to the already known immunoadhesin sequences. 4L6 and 5L7 sequences originate from recombinant anti-SIV Fab sequences derived from the bone marrow of SIV-infected rhesus monkeys[26]. Amino acid sequence alignments of 4L6 and 5L7 variable regions to the most closely corresponding rhesus genomic regions are depicted in (S9 Fig). Rhesus IgG1 sequence is based on accession no. AAF14058 and AAQ57555, and rhesus IgG2 sequence is based on AAF14060 and AAQ57567. Rhesus kappa light chain was designed using CL domain sequence from AAD02577. Synthesized fragments were then cloned into NotI site of scAAV or ssAAV vector plasmids[4]. Both 4L6 and 5L7 recognize gp120 and gp140 forms of the envelope glycoprotein.

### Rhesus IgG proteins

HEK293T cells (ATCC) were transfected with rAAV vector plasmids and proteins were purified from cell culture supernatant using Protein A Plus (Pierce). Concentration of purified proteins was determined by NanoDrop (Thermo Scientific) A_280_ measurement and purity and integrity was verified by Coomassie staining (Life Technologies).

### Binding assay

Immunoadhesins and mAbs were tested for their ability to bind SIVmac239 envelope glycoprotein by ELISA. Test plates were coated with recombinant gp140 of SIVmac239 (Immune Technology) for 1 h at 37°C. Plates were washed using PBS-Tween20 (Sigma-Aldrich) and subsequently blocked with 5% nonfat dry milk in PBS (Bio-Rad). Immunoadhesins, mAbs, positive and negative sera from macaques were serially diluted 1:3 in blocking buffer and added to the test plate. After 1 h of incubation at 37°C the plates were washed again and a HRP-conjugated goat anti-rhesus IgG H+L (SouthernBiotech) was then added for detection. The reaction was stopped after 1 h at 37°C and plates were washed 10 times. Subsequently, TMB substrate and stop solutions (SouthernBiotech) were added and Absorbance at 450 nm was measured in a microplate reader (PerkinElmer).

### Virus neutralization assay

Neutralization activity of mAbs against tested SIV strains was measured by either a luciferase-based assay as described previously (TZMbl)[36] or an assay that is based on secreted alkaline phosphatase (SEAP)[36]. Virus and a serial dilution of Abs or serum were incubated for 1 h at 37°C before adding it to the TZMbl or SEAP cells (ATCC). Neutralization was assessed two days later by measuring luminescence in a 96-well plate reader (Perkin Elmer).

### ADCC assay

Measurement of antibody effector function was performed by NK cell activity towards virus-infected target cells expressing luciferase[36]. A CD4+, CCR5+ T-cell line was infected with SIV four days prior assay readout. These target cells were washed three times on the day of the assay and co-cultured with a NK cell line expressing rhesus CD16 and a serial dilution of mAb or heat-inactivated serum for 8 to 10 hours. Cells were lysed afterwards and ADCC was measured as the loss of luciferase activity in the cell culture supernatant.

### Recombinant AAV

Production of rAAV was conducted as described previously[65]. In short, HEK 293 cells were transfected with rAAV vector plasmid and two helper plasmids to allow generation of infectious AAV particles. After harvesting transfected cells and cell culture supernatant, rAAV was purified by three sequential CsCl centrifugation steps. Vector genome number was assessed by Real-Time PCR, and the purity of the preparation was verified by electron microscopy and silver-stained SDS-PAGE.

### Animals and AAV immunization

The animals in our study were rhesus macaques of Indian-origin (*Macaca mulatta*). We purchased the 18 monkeys and housed them at the New England Primate Research Center of Harvard Medical School in a biocontainment facility in accordance with standards of the Association for Assessment and Accreditation of Laboratory Animal Care and the Harvard Medical School Animal Care and Use Committee. The animal samples used here were collected under experimental protocols approved by the Harvard Medical Area Standing Committee on Animals, and conducted in accordance to the *Guide for the Care and Use of Laboratory Animals*. All macaques were tested negative for the presence of antibodies to SIV and AAV1 capsid prior AAV administration. The weights of the animals ranged from 2.5 kg to 17.2 kg at the time of immunization. The six 5L7 IgG1 animals were split in two groups and each animal received a total of 1.6 x 10^13^ AAV vector genomes. One group received 1.6 x 10^13^ particles of ssAAV expressing the heavy and light chains of 5L7 IgG1, the other group received 0.8 x 10^13^ particles of scAAV expressing the heavy chain of 5L7 IgG1 plus 0.8 x 10^13^ particles of the scAAV expressing the kappa light chain of 5L7 IgG1. AAV administration was conducted by four equal and deep intramuscular injections, where each animal received two separate 0.5 ml injections into both quadriceps. All 6 animals that received 4L6 IgG1 were given a total of 2.5 x 10^13^ AAV vector genomes per monkey.

### Ethics statement

The animal management program of Harvard Medical School is accredited by the American Association for the Accreditation of Laboratory Animal Care, and meets National Institutes of Health standards as set forth in the *Guide for the Care and Use of Laboratory Animals* (DHHS Publication No. (NIH)85-23 Revised 1985). The Institute also accepts as mandatory the PHS *Policy on Humane Care and Use of Laboratory Animals by Awardee Institutions* and NIH *Principles for the Utilization and Care of Vertebrate Animals Used in Testing*, *Research*, *and Training*. There is on file with the Office for Protection from Research Risks an approved Assurance of Compliance.

Animal facilities were administrated by the state and met Harvard standards for humane care and use of animals through a program of veterinary care, inspection and oversight. Animal care and welfare is the charge of the Committee on Animals, appointed by the Dean, consisting of 22 members comprising 4 veterinarians, 2 public representatives, and 16 doctoral level representatives of principal sites of animal use by Harvard Faculty. Additionally, local facilities are guided and monitored in daily activities by 8 departmental animal use committees. The procedure to avoid unnecessary discomfort, pain, or injury to animals are those prescribed in the aforementioned NIH “Guide” and additional detailed protocols for anesthesia, analgesia, tranquilization, euthanasia, or restraint have been developed and circulated by the Committee on Animals.

Rhesus macaques on study were housed in the biocontainment facility of the New England Primate Research Center of Harvard Medical School under approved protocol 04655 of the Institutional Animal Care and Use Committee (IACUC) of Harvard Medical School. The Harvard Office of Microbiological Safety oversaw the design and construction of this facility. It includes individual Hepa-filtered caging for monkeys and additional features such as a pass-through autoclave, footpedal-operated sink, and a special safety cabinet for surgical and necropsy procedures. The facility has restricted access. Individuals entering the room must suit-up with disposable cap, mask, coverall, gloves and shoe covers. Procedures used in the care and feeding of these animals are posted on the entry door. The animals were provided ad lib access to municipal source water, offered commercial monkey chow twice daily, and offered fresh produce a minimum of three times weekly. Light cycle was controlled at 12/12 hours daily. Containment facilities and procedures for macaques experimentally infected with RRV and SIV have been reviewed and approved by the Harvard Committee on Microbiological Safety.

Animals housed in the biocontainment facilities received a daily health check by both animal care technicians and veterinary professional staff. All animals received a complete physical examination on the average of once every four to six weeks. A comprehensive environmental enrichment and psychological well-being plan was in place for primates in the described studies and is available for inspection by the United States Animal and Plant Health Inspection Service (APHIS) and to officials of any pertinent organization. Euthanasia took place at defined experimental endpoints using protocols consistent with the American Veterinary Medical Association (AVMA) guidelines. Animals were first sedated with intramuscular ketamine hydrochloride at 20 mg/kg body followed by sodium pentobarbital (≥100 mg/kg) intravenously to achieve euthanasia.

### Serum concentration of AAV-delivered mAb

To measure the concentration of 5L7 IgG1 and 4L6 IgG1 *in vivo* we performed a SIVmac239 gp140 (Immune Tech)/anti-rhesus IgG ELISA (Southern Biotech). Absorbance at 450 nm was compared to a serial dilution of purified mAb produced in HEK 293T cells, and the amount of antibody in serum was extrapolated based on the mAb standard curve. Reference protein was quantified by NanoDrop (Thermo Scientific) A_280_ measurement and purity was verified by Coomassie staining (Life Technologies). Levels of AAV-delivered antibody were measured up to the time of SIV challenge: 44 weeks (5L7 IgG1) and 14 weeks (4L6 IgG1) after AAV administration. The appearance of anti-env antibody responses following SIV infection obviated our ability to measure levels of delivered mAb at post-infection time points.

### Anti-anti response

Humoral responses to the AAV-delivered mAb were measured by an antibody capture ELISA. Plates were coated with purified 5L7 IgG1 and 4L6 IgG1. After coating and blocking, we incubated the plates with antisera from the AAV-immunized monkeys. For detection, we probed with a HRP-conjugated anti-human lambda light chain antibody (Southern Biotech). This secondary antibody did not cross-react with the coated mAb on the plates since 5L7 IgG1 and 4L6 IgG1 harbor a kappa light chain. For detecting kappa anti-anti responses we produced recombinant versions of mAb that harbor a lambda light chain and detected kappa chain anti-mAb antibodies by probing with a HRP-conjugated anti-human kappa light chain antibody (Southern Biotech). To assess the target of the anti-anti response we tested the reactivity of antisera against AAV-delivered mAb and its immunoadhesin version as well as against antibody or immunoadhesin that was not given to one group of animals. For example, reactivity of antisera from 5L7 IgG1 recipient was tested against purified 5L7 IgG1, 5L7 immunoadhesin, 4L6 IgG1 and 4L6 immunoadhesin.

### SIVmac239 challenge and viral load measurement

Infectious SIVmac239 was produced in PBMCs as described previously [27]. SIV challenges were conducted by repeated low dose intravenous injection with SIVmac239. The challenge dose was 1x AID50 for the first four injection and was elevated to 2x AID50 and 10x AID50 for the fifth and sixth challenge, respectively. The animals were exposed to SIV every three weeks or until positive viremia was measured. Challenge virus in plasma was detected by quantitative real-time RT-PCR using primers specific for SIV gag as described previously[66].

### Reactivity of serum to gp41

Presence or absence of anti-SIV envelope antibodies following SIV challenge was assessed by measuring the reactivity of sera (before and after challenge) against purified SIVmac251 truncated gp41 protein (ImmunoDX) by ELISA.

## Supporting Information

S1 Fig Schematic illustration of vector design and biosynthesis of full-length antibodies. Two strategies are used to generate authentic IgGs delivered by recombinant adeno-associated virus. (**Top**) In the two vector approach heavy (H) and light (L) chains of immunoglobulins are expressed from separate self-complementary adeno-associated virus vectors (scAAV). The expression cassettes consisting of a short CMV enhancer/CMV promoter, SV40 intron, rhesus macaque immunoglobulin G (IgG) heavy chain or light chain coding sequences (CDS) and polyA signal are flanked by AAV serotype 2 inverted terminal repeats (ITR). The 3' ITR of the scAAV vector has the wild-type (wt) sequence, whereas the 5' ITR is mutated lacking the wild-type terminal resolution site (trs). Heavy and light chains are individually transcribed and subsequently translated into the lumen of the endoplasmic reticulum (ER) where they co-translationally fold, undergo N-glycosylation and finally assemble. After further modifications in the Golgi apparatus authentic IgGs are secreted. (**Bottom**) To ensure delivery of heavy and light chains into the cells at equimolar levels, both polypeptides are expressed from one open reading frame using a F2A self-processing peptide from foot-and-mouth disease virus. The bicistronic single-stranded adeno-associated virus (ssAAV) construct consists of CMV promoter, SV40 intron, heavy and light chain coding sequences (CDS), separated by F2A, SGSG, Furin peptide and polyA signal flanked by AAV2 wt ITRs. In the one vector approach both heavy and light chain are transcribed from a single mRNA sequence that is subsequently translated into the ER lumen. While being elongated, the polyprotein sequence undergoes self-cleavage through a ribosomal 'skip' mechanism when reaching amino acid residues of F2A. Then, the two polypeptide chains are folded, glycosylated and assemble within the lumen of the ER. Further modifications in the Golgi lead to removal of F2A peptide residues induced by Furin cleavage. Carboxypeptidases then remove redundant Furin residues. Full-length, authentic IgGs are finally secreted.
(TIF)

S2 Fig Overlay of lambda and kappa responses to 5L7 IgG1 with levels of 5L7 IgG1 in serum. Concentration of 5L7 IgG1 expressed in μg/ml (in black) was overlaid with lambda anti-5L7 responses (in red) and kappa anti-5L7 (in blue), both expressed as absorbance at 450 nm in an ELISA. Each individual panel represents one monkey from the 5L7 mAb group.
(TIF)

S3 Fig Overlay of lambda and kappa responses to 4L6 IgG1 with levels of 4L6 IgG1 in serum. Concentration of 4L6 IgG1 expressed in μg/ml (in black) was overlaid with lambda anti-4L6 responses (in red) and kappa anti-4L6 (in blue), both expressed as absorbance at 450 nm in an ELISA. Each individual panel represents one monkey from the 4L6 mAb group.
(TIF)

S4 Fig Reactivity of serum against SIV gp41. Pre-challenge sera (in blue bars) and sera 11 weeks post-infectious exposure (9 weeks after 10x challenge for 84–05; in red bars) were tested by ELISA against SIV gp41 recombinant protein.
(TIF)

S5 Fig Analysis of peak viremia and mAb levels in the 5L7 mAb group. A Pearson’s correlation test was conducted for the average levels of 5L7 IgG1 in serum (weeks 10–44) and viral loads at peak height. The respective animal identification numbers are included in the graph. Animal 84–05 was excluded since it remained uninfected. Animals with the lowest levels of 5L7 mAb had the highest peak viral loads; the results were not statistically significant (P = 0.0658).
(TIF)

S6 Fig Comparison of 4L6 mAb levels to the time of peak viremia. Levels of 4L6 IgG1 in serum were measured on the day of infectious exposure and compared to the time of peak viremia in the 4L6 mAb group. Animal identification numbers are included in the graph with their respective effective infectious SIV exposure. Animals that displayed SIV peak viremia at week 3 had no significantly higher levels of 4L6 IgG1 at the time of infectious exposure (P = 0.4433).
(TIF)

S7 Fig Lack of ADCC-enhancing activity in pre-AAV serum from 84–05. Pre-AAV serum (week -1) from animal 84–05 was tested for potential ADCC activity against SIVmac239 infected target cells. Test sera were compared to ADCC of purified 5L7 IgG1 produced in 293T cells (84.2 μg/ml corresponds to the serum conc. of 5L7 IgG1 at week 21 post AAV administration). Purified 5L7 IgG1 was added to the pre-AAV serum from 84–05 and included in this assay. Pre-AAV serum not only had no detectable ADCC activity, it did not have any ADCC-enhancing activity when added to purified 5L7 IgG1.
(TIF)

S8 Fig ADCC activity of week 24 sera and purified 5L7 IgG1 against SIVmac239-infected target cells. ADCC was measured by the luciferase activity in SIV-infected cells after a 10 h incubation in the presence of a macaque CD16^+^ NK cell line and a serial dilution of antibodies or animal sera. The loss of RLU indicates the loss of virus-infected cells during the 10 h incubation period and represents a high ADCC activity. Purified 5L7 IgG1 was diluted to match the equivalent 5L7 IgG1 concentration of 5L7 IgG1-containing animal sera from week 24 post AAV administration, and compared for mediating ADCC towards SIV-infected target cells. Each monkey is plotted separately (serum and corresponding mAb), while the same control sera is used in each individual panel for comparison.
(TIF)

S9 Fig Amino acid sequence alignments of 4L6 and 5L7 variable regions to the most closely corresponding rhesus genomic regions. Variable regions of heavy chain (VH) and light chain (VL, kappa) of the mAbs 4L6 and 5L7 were compared to the rhesus monkey immunoglobulin germline sequences using the IMGT/V-QUEST and IMGT/BLAST analysis tools (available through http://www.imgt.org). Comparison of variable regions was conducted based on sequence identity with the V-gene repertoire (IGHV and IGKV) of Macaca mulatta; CDR3 and adjacent FR4 germline sequences were not depicted at full-length since the complete segments of those sequences are created from recombination with D-genes and J-genes. Identical amino acids are shaded in grey, framework regions (FR) and hypervariable complementarity-determining regions (CDR) are indicated by colored arrows. Corresponding V-genes and alleles were selected based on highest sequence identity with the 4L6 and 5L7 variable regions.
(TIF)

S1 Table Details on monkeys used in the study. (TIF)
