# Supplementary figures and images for: AAV-Delivered Antibody Mediates Significant Protective Effects against SIVmac239 Challenge in the Absence of Neutralizing Activity
Source: PLoS Pathog. 2015 Aug 6;11(8):e1005090. doi: 10.1371/journal.ppat.1005090 (PMC4527674; doi:10.1371/journal.ppat.1005090)

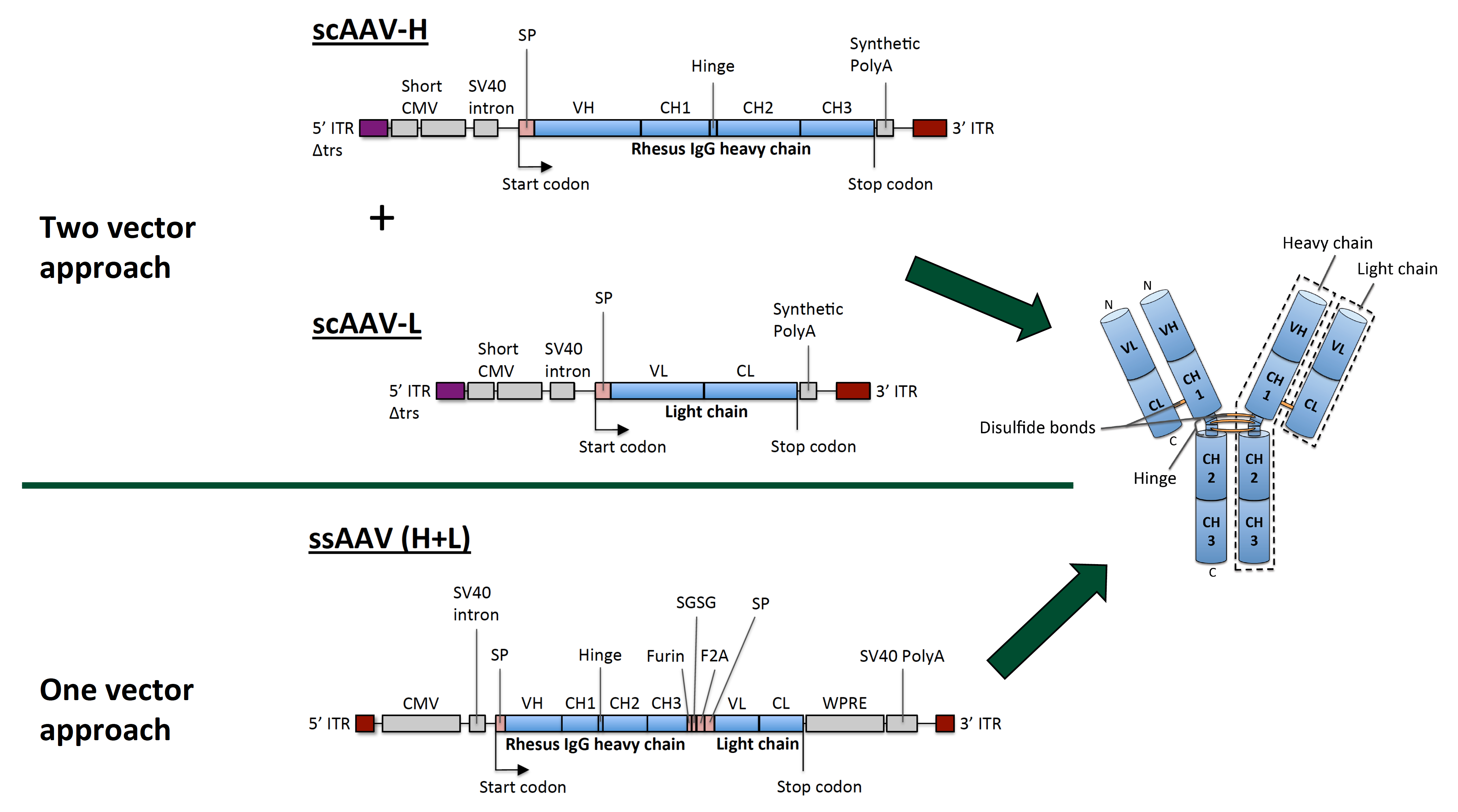

Supplement: S1 Fig — Two strategies are used to generate authentic IgGs delivered by recombinant adeno-associated virus. (Top) In the two vector approach heavy (H) and light (L) chains of immunoglobulins are expressed from separate self-complementary adeno-associated virus vectors (scAAV). The expression cassettes consisting of a short CMV enhancer/CMV promoter, SV40 intron, rhesus macaque immunoglobulin G (IgG) heavy chain or light chain coding sequences (CDS) and polyA signal are flanked by AAV serotype 2 inverted terminal repeats (ITR). The 3' ITR of the scAAV vector has the wild-type (wt) sequence, whereas the 5' ITR is mutated lacking the wild-type terminal resolution site (trs). Heavy and light chains are individually transcribed and subsequently translated into the lumen of the endoplasmic reticulum (ER) where they co-translationally fold, undergo N-glycosylation and finally assemble. After further modifications in the Golgi apparatus authentic IgGs are secreted. (Bottom) To ensure delivery of heavy and light chains into the cells at equimolar levels, both polypeptides are expressed from one open reading frame using a F2A self-processing peptide from foot-and-mouth disease virus. The bicistronic single-stranded adeno-associated virus (ssAAV) construct consists of CMV promoter, SV40 intron, heavy and light chain coding sequences (CDS), separated by F2A, SGSG, Furin peptide and polyA signal flanked by AAV2 wt ITRs. In the one vector approach both heavy and light chain are transcribed from a single mRNA sequence that is subsequently translated into the ER lumen. While being elongated, the polyprotein sequence undergoes self-cleavage through a ribosomal 'skip' mechanism when reaching amino acid residues of F2A. Then, the two polypeptide chains are folded, glycosylated and assemble within the lumen of the ER. Further modifications in the Golgi lead to removal of F2A peptide residues induced by Furin cleavage. Carboxypeptidases then remove redundant Furin residues. Full-length, authent [file ppat.1005090.s001.tif]

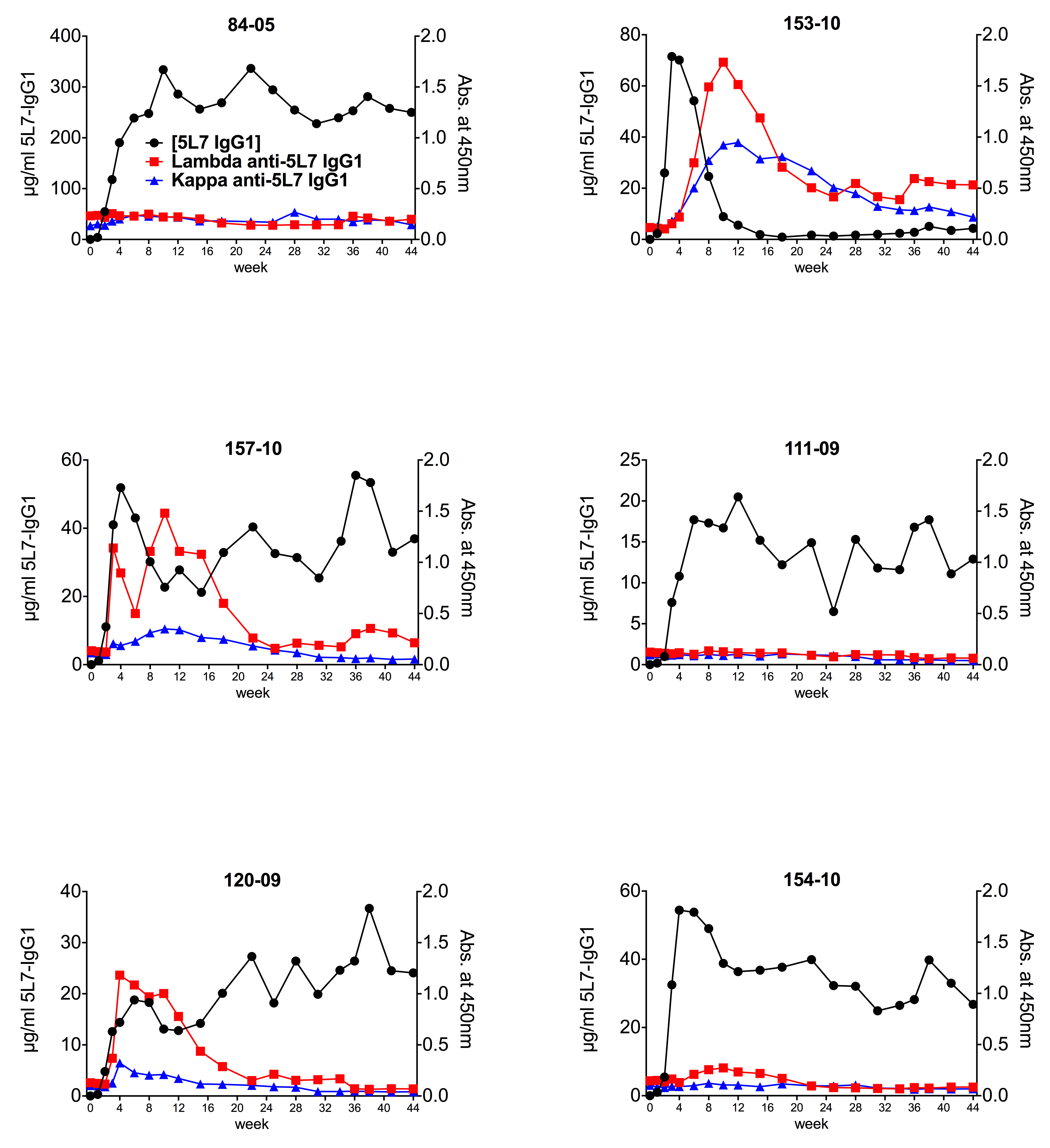

Supplement: S2 Fig — Concentration of 5L7 IgG1 expressed in μg/ml (in black) was overlaid with lambda anti-5L7 responses (in red) and kappa anti-5L7 (in blue), both expressed as absorbance at 450 nm in an ELISA. Each individual panel represents one monkey from the 5L7 mAb group. (TIF) [file ppat.1005090.s002.tif]

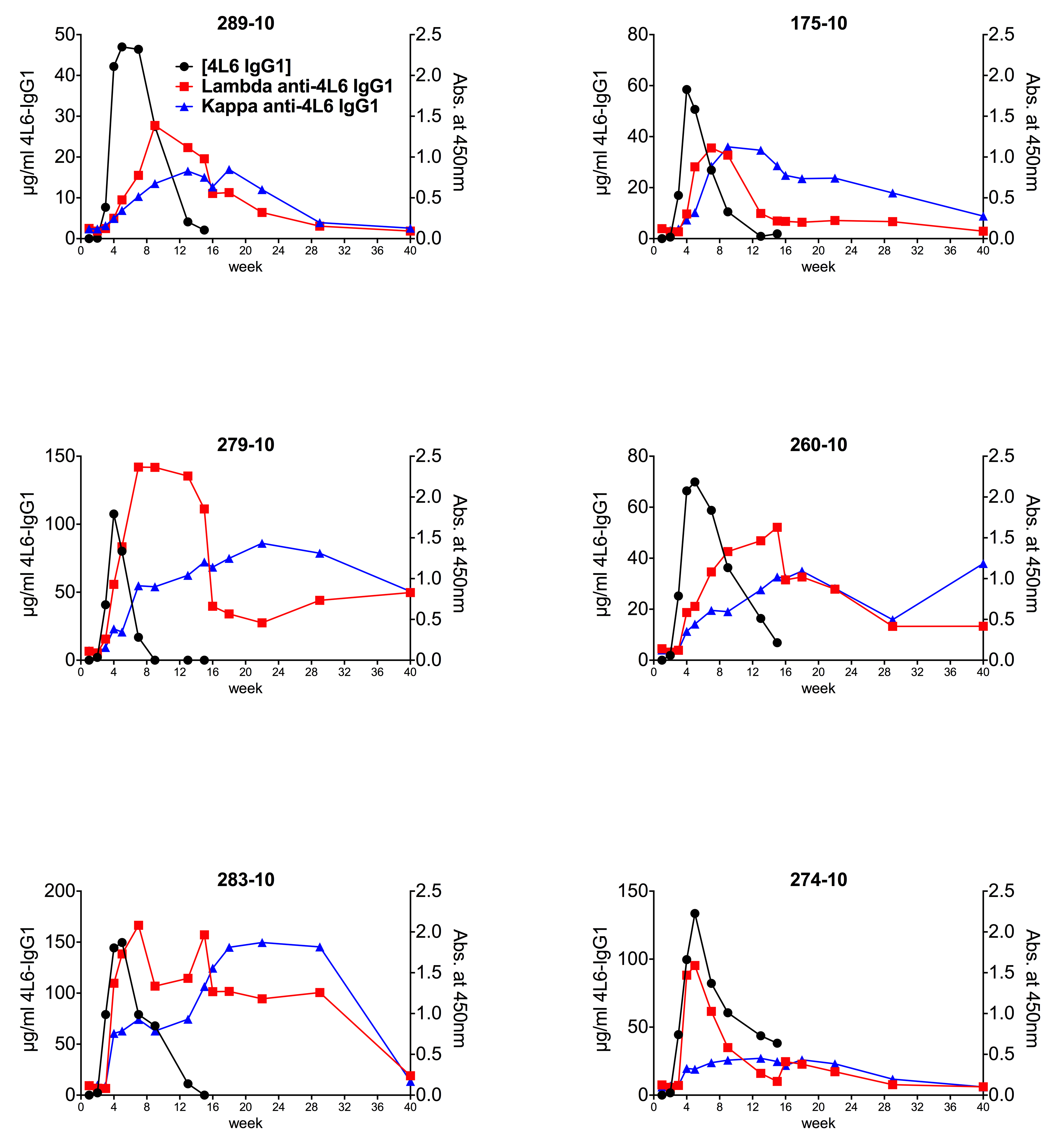

Supplement: S3 Fig — Concentration of 4L6 IgG1 expressed in μg/ml (in black) was overlaid with lambda anti-4L6 responses (in red) and kappa anti-4L6 (in blue), both expressed as absorbance at 450 nm in an ELISA. Each individual panel represents one monkey from the 4L6 mAb group. (TIF) [file ppat.1005090.s003.tif]

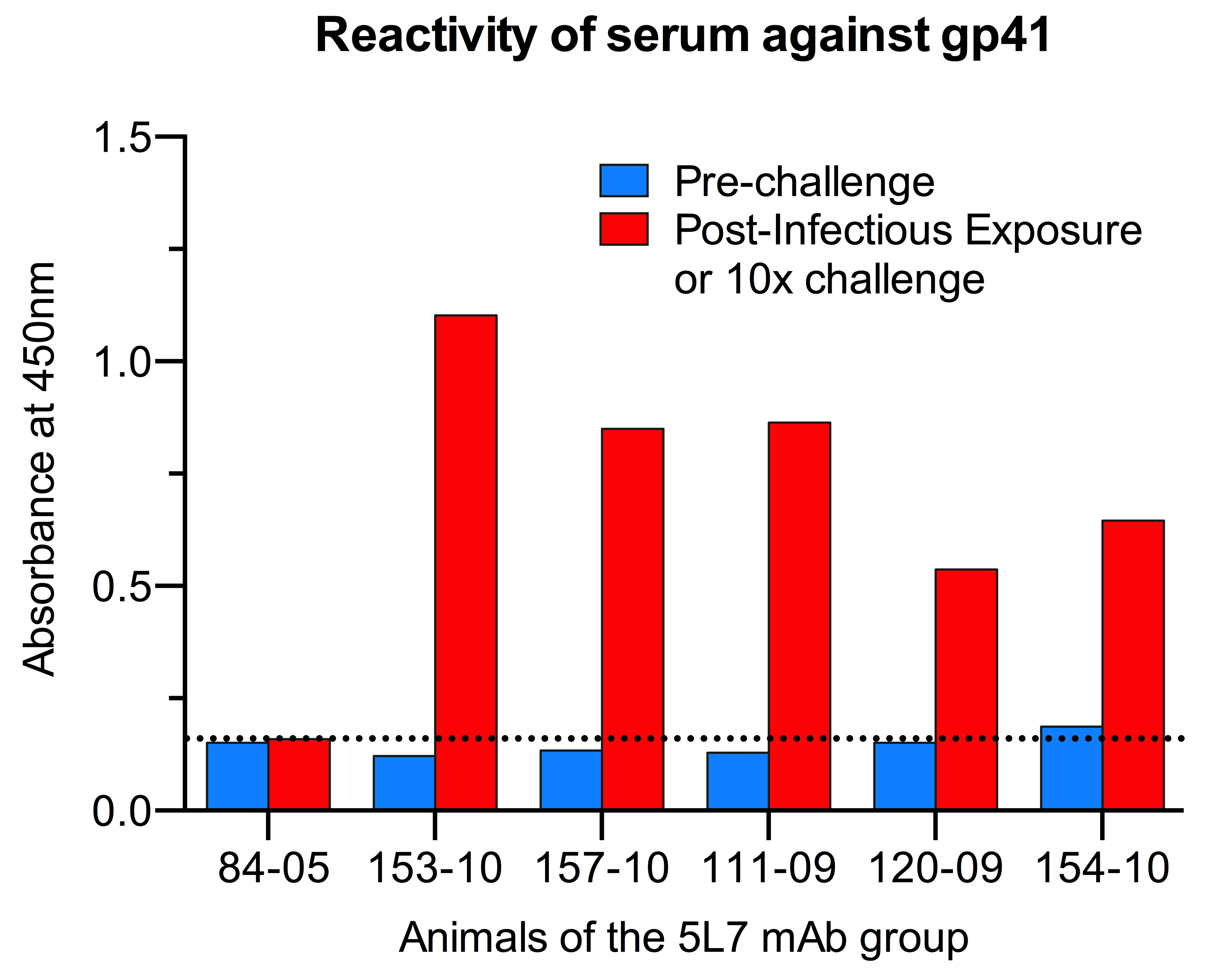

Supplement: S4 Fig — Pre-challenge sera (in blue bars) and sera 11 weeks post-infectious exposure (9 weeks after 10x challenge for 84–05; in red bars) were tested by ELISA against SIV gp41 recombinant protein. (TIF) [file ppat.1005090.s004.tif]

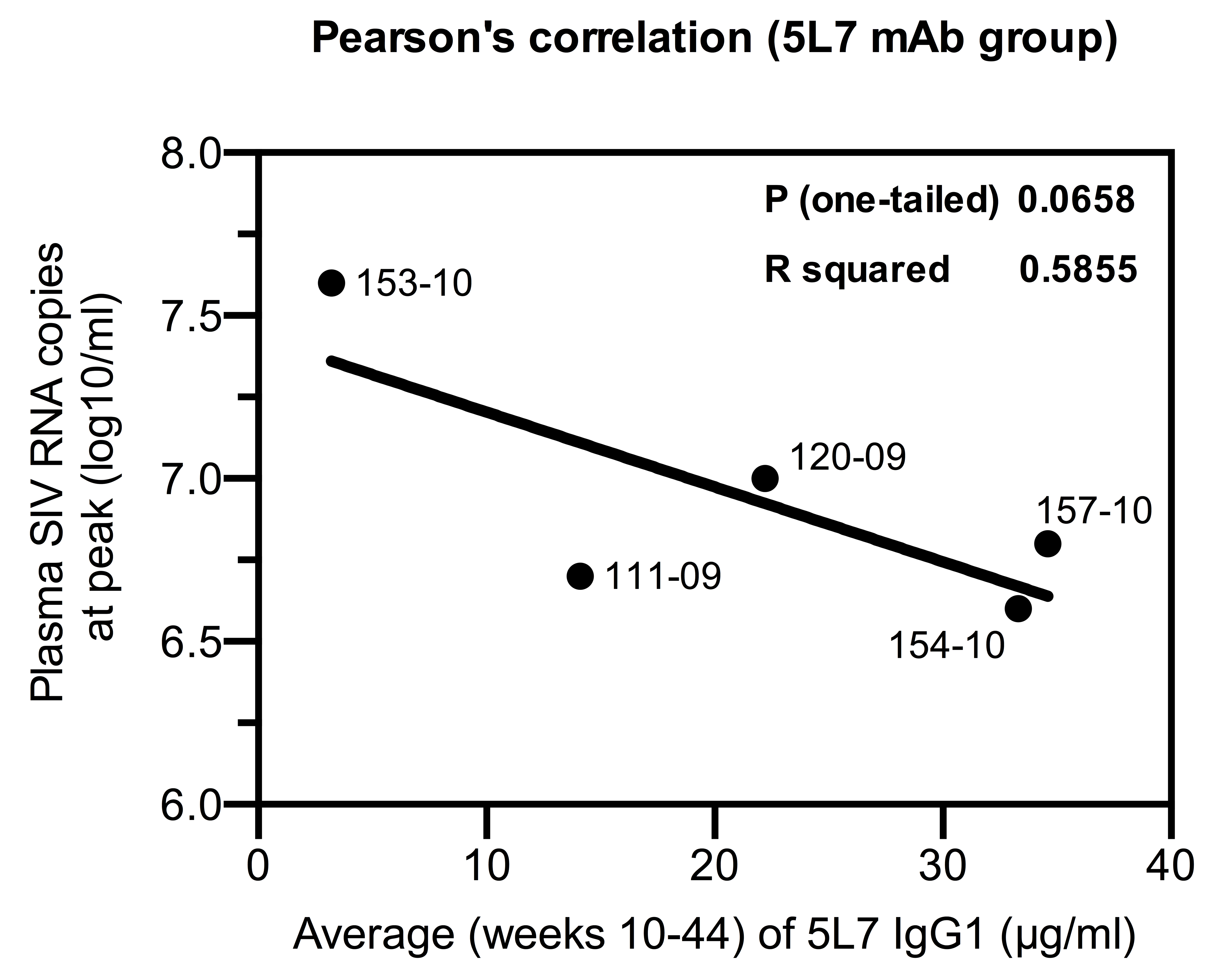

Supplement: S5 Fig — A Pearson’s correlation test was conducted for the average levels of 5L7 IgG1 in serum (weeks 10–44) and viral loads at peak height. The respective animal identification numbers are included in the graph. Animal 84–05 was excluded since it remained uninfected. Animals with the lowest levels of 5L7 mAb had the highest peak viral loads; the results were not statistically significant (P = 0.0658). (TIF) [file ppat.1005090.s005.tif]

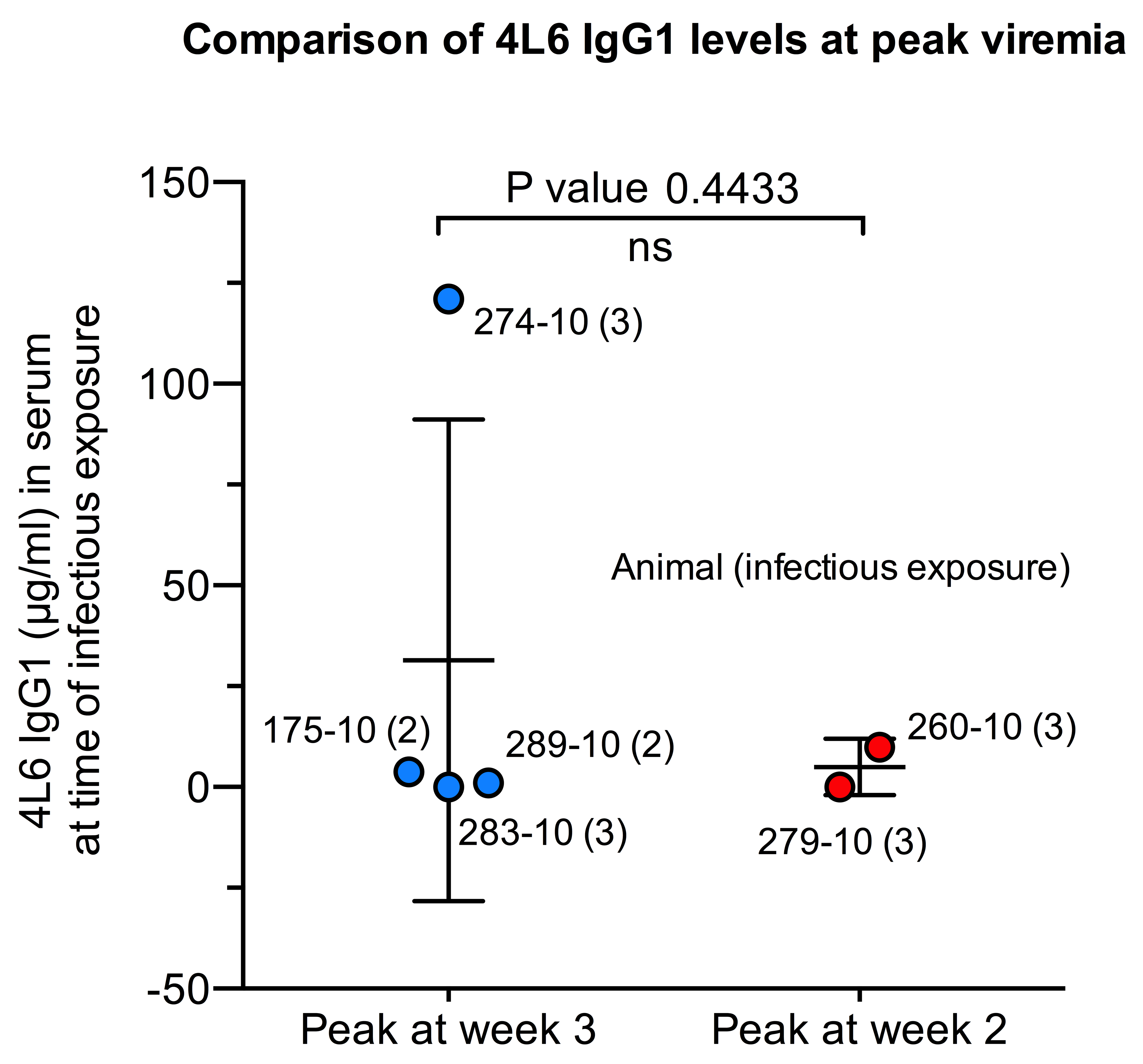

Supplement: S6 Fig — Levels of 4L6 IgG1 in serum were measured on the day of infectious exposure and compared to the time of peak viremia in the 4L6 mAb group. Animal identification numbers are included in the graph with their respective effective infectious SIV exposure. Animals that displayed SIV peak viremia at week 3 had no significantly higher levels of 4L6 IgG1 at the time of infectious exposure (P = 0.4433). (TIF) [file ppat.1005090.s006.tif]

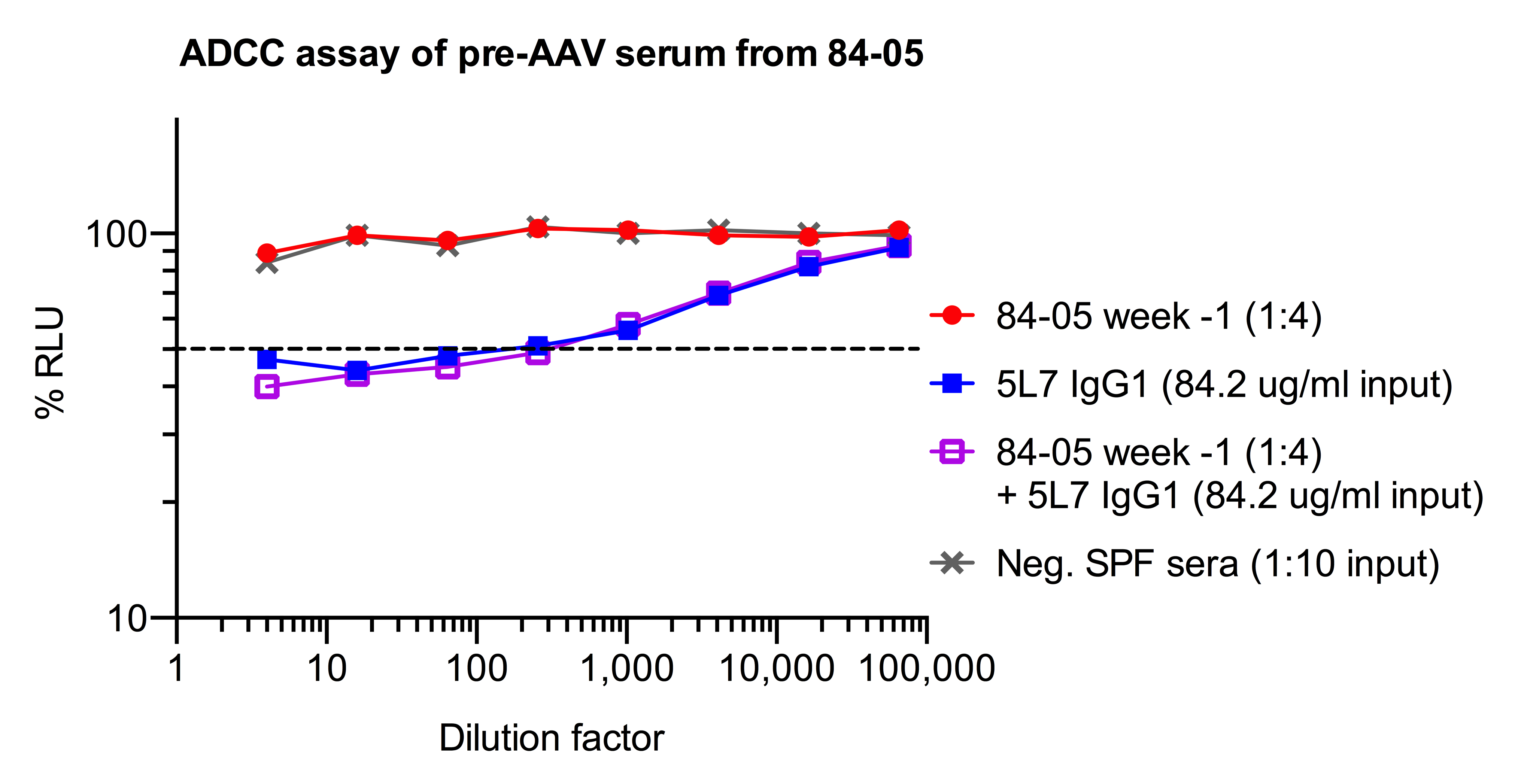

Supplement: S7 Fig — Pre-AAV serum (week -1) from animal 84–05 was tested for potential ADCC activity against SIVmac239 infected target cells. Test sera were compared to ADCC of purified 5L7 IgG1 produced in 293T cells (84.2 μg/ml corresponds to the serum conc. of 5L7 IgG1 at week 21 post AAV administration). Purified 5L7 IgG1 was added to the pre-AAV serum from 84–05 and included in this assay. Pre-AAV serum not only had no detectable ADCC activity, it did not have any ADCC-enhancing activity when added to purified 5L7 IgG1. (TIF) [file ppat.1005090.s007.tif]

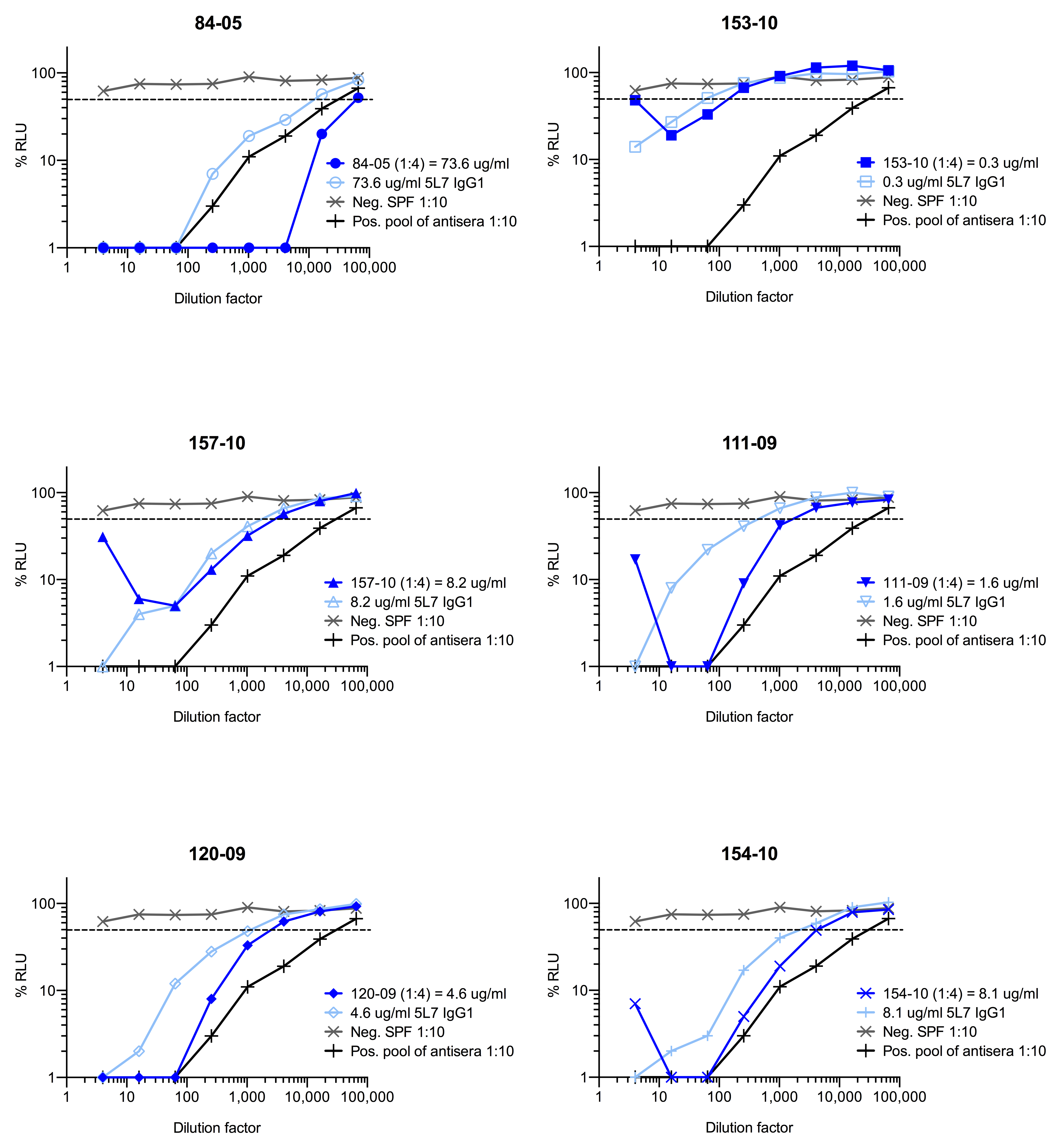

Supplement: S8 Fig — ADCC was measured by the luciferase activity in SIV-infected cells after a 10 h incubation in the presence of a macaque CD16+ NK cell line and a serial dilution of antibodies or animal sera. The loss of RLU indicates the loss of virus-infected cells during the 10 h incubation period and represents a high ADCC activity. Purified 5L7 IgG1 was diluted to match the equivalent 5L7 IgG1 concentration of 5L7 IgG1-containing animal sera from week 24 post AAV administration, and compared for mediating ADCC towards SIV-infected target cells. Each monkey is plotted separately (serum and corresponding mAb), while the same control sera is used in each individual panel for comparison. (TIF) [file ppat.1005090.s008.tif]

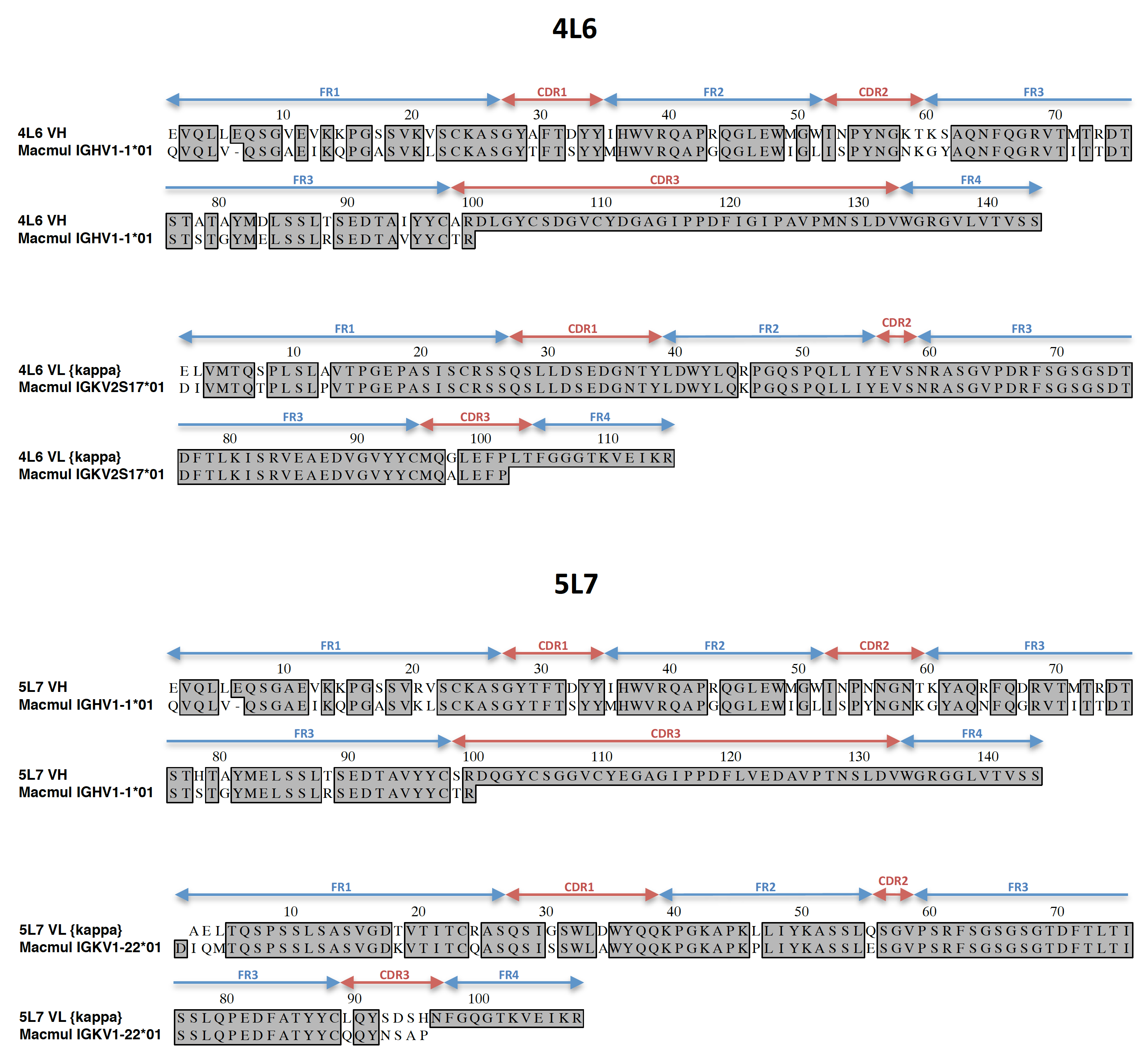

Supplement: S9 Fig — Variable regions of heavy chain (VH) and light chain (VL, kappa) of the mAbs 4L6 and 5L7 were compared to the rhesus monkey immunoglobulin germline sequences using the IMGT/V-QUEST and IMGT/BLAST analysis tools (available through http://www.imgt.org). Comparison of variable regions was conducted based on sequence identity with the V-gene repertoire (IGHV and IGKV) of Macaca mulatta; CDR3 and adjacent FR4 germline sequences were not depicted at full-length since the complete segments of those sequences are created from recombination with D-genes and J-genes. Identical amino acids are shaded in grey, framework regions (FR) and hypervariable complementarity-determining regions (CDR) are indicated by colored arrows. Corresponding V-genes and alleles were selected based on highest sequence identity with the 4L6 and 5L7 variable regions. (TIF) [file ppat.1005090.s009.tif]

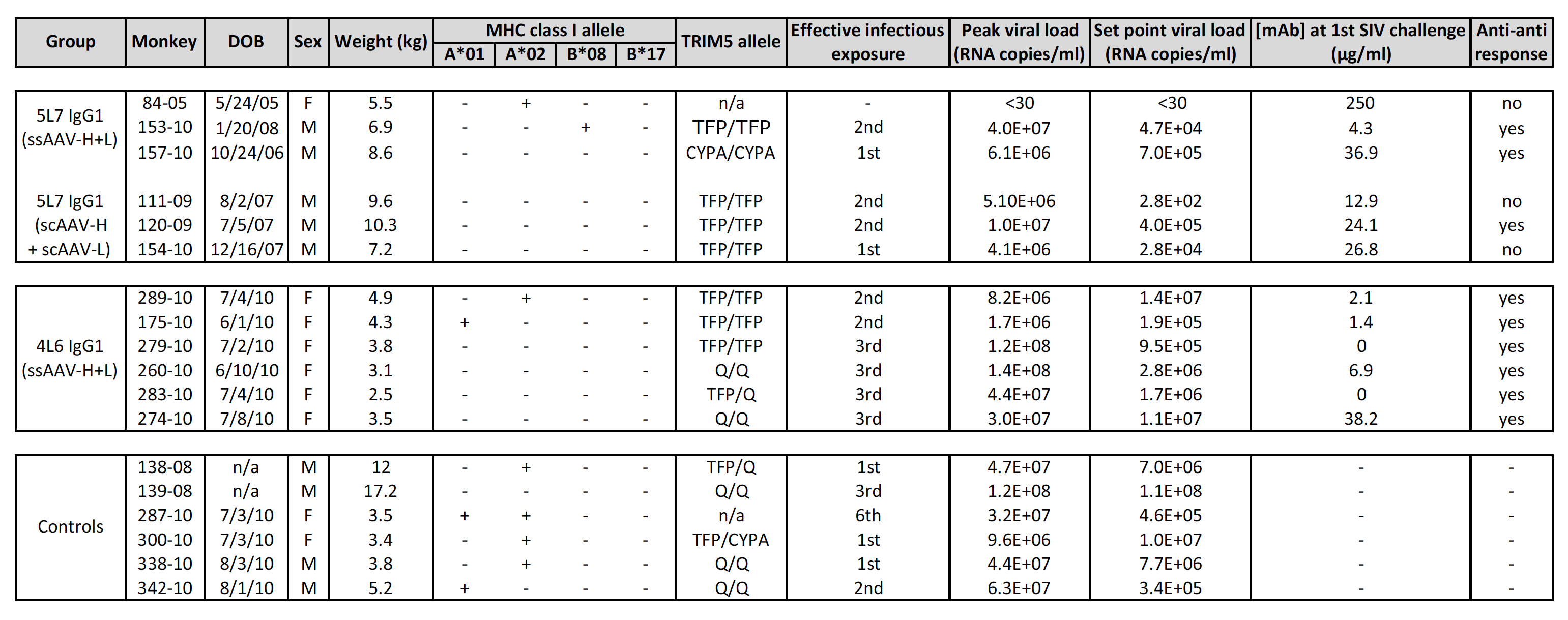

Supplement: S1 Table — (TIF) [file ppat.1005090.s010.tif]
